# Private Letter from Prof. J. P. White

**Published:** 1866-07

**Authors:** 


PRIVATE LETTER FROM PROF. J. P. WHITE.—We publish a portion of a private letter from Prof. White, knowing that many of our readers will be also greatly interested:

SORENTO, June 5th, 1866.

Dear Doctor:—If you publish the accompanying communication for the Journal, look well to the proof. I cannot find time to copy my manuscript, and necessarily write hastily and under many inconveniences. I am writing at this moment in dorento, upon the sea, near Naples, within sight of Vesuvius, and overlooking the Seautiful Bay of Naples. James is quite ill, and it is now so late, being already betained a fortnight by his illness, that we shall scarcely get to Constantinople as I had hoped. It is doubtful whether war or peace is to prevail in Europe, and the present disturbed state of the country embarrasses the traveler greatly. These and many other reasons which I might enumerate, render it highly probable that I shall shorten my proposed stay abroad, and may be home during the autumn of this year. I hear nothing from you except the letter already mentioned in Florence. My own health improves steadily. I went up Vesuvius last week, a task requiring no little physical strength.

Yours, very truly, J. P. WHITE.

---

BUFFALO, June 29, 1866.

To the Editor of the Buffalo Medical and Surgical Journal:

Dear Sir:—In the report of the proceedings of the Buffalo Medical Association, published in the June number of the Journal, I am made to say that I have "never met with inflammation of the brain with otitis." The statement I made was directly the reverse; neither did I speak of "disease of the base of the *medulla spinalis*," but of the base of the brain. There are several other errors, typographical or clerical, which would not have appeared had an opportunity been afforded me of correcting the proof. I know our reporter will say, that those who desire to have their remarks correctly given should write them out. That is true, but not always practicable. I do not complain of any omissions, but I do feel a little sensitive, when I am represented to have said what I have not uttered.

Yours, respectfully, THOS. F. ROCHESTER.

We are happy to publish the above communication from Dr. Rochester as a correction of the report of his remarks; but the "errors typographical or clerical," are in no way chargable upon the proof reader or publisher of the Journal. The copy is published as furnished and corrected by the Secretary.—EDITOR.

---

TRANSFUSION OF BLOOD.—MM. Eulenburgh and Landois have ascertained that animals poisoned by opium may be kept alive by means of a combined transfusion of blood, draining away the poisoned blood, and substituting healthy blood taken from an animal of the same species.

